# Improving the Course of Depressive Symptoms After Inpatient Psychotherapy Using Adjunct Web-Based Self-Help: Follow-Up Results of a Randomized Controlled Trial

**DOI:** 10.2196/13655

**Published:** 2019-10-24

**Authors:** Rüdiger Zwerenz, Carlotta Baumgarten, Jan Becker, Ana Tibubos, Martin Siepmann, Rudolf J Knickenberg, Manfred E Beutel

**Affiliations:** 1 Department of Psychosomatic Medicine and Psychotherapy University Medical Center Johannes Gutenberg-University Mainz Germany; 2 Institute for Teachers’ Health University Medical Center Johannes Gutenberg-University Mainz Germany; 3 Clinic for Psychosomatic Medicine Rhön-Klinikum AG Bad Neustadt/ Saale Germany; 4 Department of Psychotherapy and Psychosomatic Medicine Faculty of Medicine Carl Gustav Carus Technische Universität Dresden Dresden Germany

**Keywords:** depression, mental health, internet, aftercare, psychotherapy, psychology, clinical, inpatients

## Abstract

**Background:**

We recently showed in a randomized controlled trial that Web-based self-help as an adjunct improved the effectiveness of multimodal inpatient psychotherapy for depression.

**Objective:**

The aims of this study were (1) to determine whether a Web-based self-help adjunctive to multimodal inpatient psychotherapeutic treatment could also improve the course of depressive symptoms and (2) to identify predictors of residual depressive symptoms at follow-up.

**Methods:**

Overall, 229 patients were randomized either to the Web-based self-help intervention group (Deprexis) or an active control group (Web-based information about depression and depressive symptoms) in addition to multimodal inpatient psychotherapy. Participants in both groups were able to access their respective Web-based programs for 12 weeks, which meant that they typically had access after discharge from the inpatient unit (mean hospitalization duration: 40 days, T1). Follow-up was performed 6 months after study intake (T3).

**Results:**

At follow-up, participants of the Web-based self-help group had considerably lower symptom load regarding depressive symptoms (*d*=0.58) and anxiety (*d*=0.46) as well as a better quality of life (*d*=0.43) and self-esteem (*d*=0.31) than participants of the control group. Nearly 3 times as many participants of the intervention group compared with the control group achieved remission in accordance with less deterioration. The number needed to treat based on the Beck Depression Inventory-II (BDI-II) improved over time (T1: 7.84, T2: 7.09, and T3: 5.12). Significant outcome predictors were BDI at discharge and treatment group.

**Conclusions:**

Web-based self-help as an add-on to multimodal inpatient psychotherapy improved the short-term course of depressive symptoms beyond termination. Residual symptoms at discharge from inpatient treatment and utilization of the Web-based self-help were the major predictors of depressive symptoms at follow-up. Challenges and barriers (eg, costs, therapists’ concerns, or technical barriers) of adding Web-based interventions to inpatient treatment have to be addressed.

**Trial Registration:**

ClinicalTrials.gov NCT02196896; https://clinicaltrials.gov/ct2/show/NCT02196896.

## Introduction

### Benefit and Effectiveness of Web-Based Interventions

Depression has been recognized as one of the leading health problems with a 12-month prevalence of 6.9% [1] and an even higher prevalence of depression and depressive symptoms in outpatients of different clinical specialties (27%) [2]. Given its high prevalence and limited access to evidence-based treatments [3], Web-based self-help interventions have been developed to provide instant, flexible help for a great variety of mental health problems [4]. Several meta-analyses have shown that they are effective treatments of depressive symptoms with effect sizes comparable with face-to-face treatments [5] and have been proven to prevent successfully the recurrence in remitted depression patients [6,7]. Efficacy, however, depends on contextual factors Web-based interventions are implemented, such as a meta-analysis [8] of the Web-based intervention moodgym showed. Sensitivity analyses have shown that general efficacy (*g*=0.36) is lower (*g*=0.17), when publication-bias is considered, an active control group is used as a comparative condition (*g*=0.12 vs *g*=0.53), guidance is missing (*g*=0.23 vs *g*=0.75), or adherence is low (*g*=0.22 vs *g*=0.64); on the other hand efficacy is higher in some countries, such as Australia (*g*=0.78) than in others (eg, Europe *g*=0.17).

Deprexis is an interactive Web-based self-help program [9] with proven effectiveness in several randomized controlled studies (RCTs). In a recent meta-analysis of self-guided Web-based interventions for the treatment of depressive symptoms [10], 13 trials of which 5 were Deprexis trials have been combined, and the overall effect was *g*=0.31. Compared with the meta-analysis of Karyotaki [10] with heterogenous internet-based cognitive behavioral therapy interventions, a recent meta-analysis based on 8 studies with Deprexis reported a slightly higher posttreatment between-group effect size for the improvement of depressive symptoms of *g*=0.54 (95% CI 0.39-0.69) [11]. Moreover, there is evidence for efficacy after completing the intervention with between-group effect size of *d*=0.32 in a 6-month follow-up compared with usual psychological or pharmacological treatments alone [12]. In another study, between-group effect sizes from *d*=0.36 at 3 months to *d*=0.13 at 12 months were reported, and remission rates over time were significantly higher in the group receiving Deprexis [13].

### Web-Based Interventions in Regular Care and Blended Treatments

However, the challenge has remained how to integrate Web-based treatments into regular care [5], especially as effectiveness studies show quite mixed results. In a large RCT of 2 Web-based cognitive behavioral interventions, which had proven to be effective in prior RCTs, no statistical significant effect could be shown when these interventions have been added to usual care [14]. In an RCT, adding an internet-based relapse prevention program to treatment as usual (TAU) was not cost-effective regarding depression-free days and quality adjusted life years compared with TAU alone [15].

In blended treatments, face-to-face interventions are combined with Web-based or mobile-based interventions and integrated in 1 treatment scheme [16]. Nakao et al [17] compared a Web-based program combined with face-to-face sessions with regular cognitive behavioral therapy in an RCT with N=40 participants and could show that blended CBT was effective in reducing depressive symptoms in patients with major depression. There are also first promising studies on blended group psychotherapy; in a qualitative study, patients reported in in-depth interviews that blended group therapy could be motivating, increasing the consolidation of the results of face-to-face psychotherapy, among other things, because of the possibility of between-session monitoring and Web-based reinforcement of exercising tasks [18]. In a randomized controlled feasibility study, large between-group effect sizes (*d*=0.87) could be shown in favor of blended group psychotherapy compared with a wait list control group [19]. However, the often-proven cost-effectiveness of certain Web-based interventions (eg, McCrone et al [20]) is not automatically the case for blended treatments. In a naturalistic study, 4448 records of patients with depressive symptoms or anxiety were compared using propensity score matching whether treatment was done in a regular face-to-face setting or blended treatment; 1 main result was that blended treatment resulted in significantly higher costs mainly because of more treatment time than in the regular face-to-face setting [21].

### Web-Based Interventions as an Add-On to Inpatient Psychotherapy

Inpatient psychotherapy is indicated in severe, chronic and complex cases of depression, compounded by mental or somatic comorbid conditions according to German medical guidelines (DGPPN 2015) [22]. It has reduced depressive symptoms with a large effect size (*d*=1.2) based on the Beck Depression Inventory (BDI) after 61.8 days of inpatient treatment in a multicenter study [23], respectively; Hedges *g*=0.84 according to a meta-analysis [24]. On the basis of the Quick Inventory of Depressive Symptoms expert rating), Zeeck et al [25] reported complete remission in 29% of patients, partial remission in 11%, modest change of 31%, and nonresponse in 29% in a multicenter study with a mean inpatient or day hospital treatment duration of 10 weeks. However, even after successful inpatient treatment, there may be residual symptoms that increase the risk of relapse or recurrence of depressive symptoms [26,27].

In an RCT with 229 depression inpatients [28], we compared Deprexis with an active control group of psychoeducation comprising a weekly Web-based information regarding etiology and treatment of depression. Both conditions were adjuncts to intensive psychodynamic inpatient psychotherapy with a mean duration of 40 days (range 11-78 days). As participants were eligible to use Deprexis for a total of 12 weeks, they could continue to use it for an average of at least 6 weeks after discharge from treatment. Depressive symptoms were significantly lower in the intervention group at discharge from inpatient treatment and at the end of intervention (3 months after study inclusion), with a moderate effect size (*d*=0.47 at discharge and *d*=0.44 3 months after study inclusion). This counted also for anxiety, quality of life, and self-esteem with effect sizes between *d*=0.33 and *d*=0.38 at discharge [28].

The aim of this study was to determine whether the add-on of a Web-based self-help program to multimodal inpatient treatment of depressed patients also improves stability of remission and test postulated predictors of residual depression [29] at follow-up based on patient characteristics. As residual symptoms were lower in the group with adjunct Web-based self-help versus psychoeducation, we hypothesized that remission rates would be higher at follow-up 6 months after study inclusion.

## Methods

### Participants

From July 2014 to February 2016, patients have been recruited in the Psychosomatic Clinic in Bad Neustadt/Saale, Germany. Eligible patients were aged between 18 to 65 years, had private internet access, sufficient German language skills, a score in the BDI-II above 13, and a clinical diagnosis of depression (International Statistical Classification of Diseases and Related Health Problems 10th revision: F32.x, F33.x, F34.1, and F43.2). Patients were excluded if a diagnosis of (1) psychosis (F20-F29); (2) current alcohol or drug addiction (F10-F19); (3) borderline (F60.3), antisocial (F60.2), schizoid (F60.1), and schizotypal (F21) personality disorders; (4) anorexia nervosa (F50.0); and (5) lifetime diagnoses of schizophrenia (F20-F29), schizoaffective (F25), bipolar (F31), or organic (F00-F09) mental disorder was present.

Eligible patients received oral and written information about the study and its requirements as part of a weekly information session. Therapists were introduced to the rationale of the intervention but had no active part in 1 of the add-on treatments.

After signing written informed consent, participants were coded and randomized to one of the groups (intervention group vs control group). Procedure and study protocol were conducted in accordance with the declaration of Helsinki and approved by the ethics committee of the Statutory Physician Board of the State of Rhineland Palatinate (Ref No 837.093.14 [9332-F]). The trial protocol was published elsewhere [30].

As described in more detail in the study by Zwerenz et al [28], 611 patients were eligible to participate in the study, 135 of whom did not meet inclusion criteria, 180 of whom did not want to participate, and 67 of whom did not complete study consent. Accordingly, out of the remaining 229 patients randomized, 215 patients were analyzed (N=108 in the intervention group and N=107 in the control group) who had received the respective intervention. At the end of inpatient treatment (T1), 198/229 (86.5%) participants completed the assessment: 87.8% (101/115) in the intervention group and 85.0% (97/114) in the control group. At the end of the intervention (T2) 74.2% (170/229) of the participants completed the assessment 73.9% (85/115) in the intervention group and 74.5% (85/114) in the control group. At the follow-up (T3), 69.9% (160/229) completed the assessment, 75.6% (87/115) in the intervention group and 64.0% (73/114) in the control group. Participants who completed the T2 and follow-up assessments did not differ from those participants who dropped out from assessments concerning baseline mental symptoms or self-assessed work ability.

Participants were predominantly female (60.7%; 139/229) and had a mean age of 48 years (SD 9.79), ranging from 18 to 65 years. About half of the participants were married (50.2%; 115/229), graduated from middle or higher secondary level (58.1%; 133/229), and worked full-time (47.6%; 109/229), with 56.3% (129/229) being on sick leave at study intake. Mean inpatient treatment duration was 40 days (range 11-78; SD 7.51), with no difference between intervention group (mean 41, SD 7.43) and control group (mean 40, SD 7.58). Previous psychopharmacological and psychotherapeutic treatments were comparable in the intervention group and control group as well as the status of antidepressant medication during inpatient treatment [28]. As delineated previously [28], the majority (79.9%; 183/229) reported having accessed the intervention or the Web-based material at least once. Furthermore, almost twice as many participants used Deprexis (46.0%; 53/115) regularly, that is, several times a week, compared with the utilization of the Web-based information in the control group (23.6%; 27/114).

### Intervention and Control Condition

In the Psychosomatic Clinic Bad Neustadt, multimodal inpatient psychodynamic psychotherapy entails individual and group psychotherapy, creative psychotherapy interventions, and adjunct treatments such as patient education and exercising. Insight-oriented group therapy was combined with nonverbal treatment methods, that is, body therapy to address difficulties in emotion modulation, interpersonal problems, core beliefs about oneself, deficits in self-esteem, and self-care that contributed to the depressive symptoms. In addition to multimodal inpatient psychodynamic psychotherapy, participants of the intervention group got access to the Web-based self-help program Deprexis for 12 weeks. It consists of 10 main modules plus an introductory and a summary module based on cognitive behavioral techniques, positive psychology, emotion-focused therapy, and dream work (for details, cf. Meyer et al [9]). An interactional dialogue guides the user presenting text blocks with optional graphics, exercises, audio files, and answering options. Subsequent text blocks are based on the user’s choices. A new module is presented only after completing the prior module. Optional reminders can be activated via email and short message service (see Meyer et al [9] and Zwerenz et al [28]). During inpatient treatment, participants had 2 1-hour time slots implemented in their weekly treatment plan, when they got access to a computer. Back home, continued access to Deprexis was provided until the period of 12 weeks had expired.

For participants of the control group, an internet platform was accessible in addition to the inpatient psychotherapy (TAU), consisting of 12 weekly modules with specific topics regarding depression, for example, information on depressive disorders, treatment options (psychotherapeutic and medication), efficacy of different treatments, and prognostic factors. This information was mainly based on the official treatment guidelines for major depression in Germany [22]. Analogous to the intervention group, the treatment plan provided participants with time slots and computer access for using the Web-based platform [28,30]. All treatments were performed by the same group of therapists.

### Outcome Measures

In addition to sociodemographic variables and relevant treatment data (ie, previous treatment, diagnosis, medication, treatment duration, etc), standardized questionnaires were used. Primary and secondary outcome measures were collected by self-report using the Web-based survey platform SoSci Survey (SoSci Survey GmbH [31]).

Depressive symptoms, our primary study outcome measured by the BDI-II [30], a reliable and valid instrument [32,33], were assessed at baseline (T0), discharge from the hospital (T1), termination of the program (T2), and follow-up (T3) 6 months after study admission. Apart from the time of discharge from the clinic (T1), secondary outcomes were also surveyed.

Secondary outcomes were assessed by well-established, reliable, and valid measures. Depressive symptoms were additionally measured with the Patient Health Questionnaire-9 (PHQ-9 [34]; Cronbach alpha=.85). Generalized anxiety was assessed with the generalized anxiety disorder-7 (GAD-7 [35]; Cronbach alpha=.92), quality of life (assessed with the European Health Interview Survey Quality of Life 8-Item index, EUROHIS-QOL 8-item index [36]; Cronbach alpha=.78), and self-esteem by the Rosenberg Self-esteem Scale (RSE [37]; Cronbach alpha=.84). Dysfunctional attitudes related to depressive thinking were measured with the Dysfunctional Attitudes Scale (DAS [38]; Cronbach alpha=.86) and work ability by the short version of the Work Ability Index (WAI [39]; Cronbach alpha=.80). Satisfaction, positive and negative influence, as well as adherence to the Web-based interventions were measured by single items on 5-point Likert scales. As potential predictor of outcome, childhood trauma was assessed with the German version of the Childhood Trauma Questionnaire [40] (Cronbach alpha=.89). To measure structural psychological deficits, the valid short form of the Operationalized Psychodynamic Diagnosis structured questionnaire [41] was used (Cronbach alpha=.61 and .87).

### Data Analyses

All analyses were performed on the basis of intention to treat. To replace missing values, we used a Markov Chain Monte Carlo multivariate imputation algorithm with IBM SPSS Statistics 23 and 5 imputations, 10 estimations per missing value, and a constraint of a maximum of 60% missing data.

Outcome measures were evaluated in both conditions by analyses of covariance with baseline scores as covariate. Remission rates, significant reliable change [42], and number needed to treat (NNT) were calculated. As a reversal of the relative risk, the NNT indicates the number of patients that have to be treated to generate an additional positive outcome in 1 of them [43]. Comparisons of the between-group effects with regard to these variables were tested with χ^*2*^ tests. To test the postulated predictors of depressive symptoms at follow-up [30], we performed multivariate analysis using a generalized linear model (GLM). According to protocol [30], depressive symptoms at baseline, childhood trauma, degree of structural deficits, and utilization of other (psychotherapeutic, pharmacological, inpatient, and self-help) treatments after the inpatient treatment were included as independent variables controlling for sex and age. Group (intervention group vs control group) was also included to evaluate the effect of each treatment. Residual depressive symptoms at discharge and its interaction with the study group were also added. To detect remission rates, we calculated the Reliable Change Index (RCI; based on the approach of Jacobson and Truax [42]). The RCI was calculated for the change of the primary outcome (BDI-II) between baseline (T0) and the respective follow-ups (T1, T2, or T3) for both groups (intervention group and control group) as follows: (1) Remission was defined as a BDI-II reduction of at least 8 and a total score below 14 (=normal range). (2) Improved but not recovered was defined as a BDI-II score reduction of at least 8 points but a total score above 14. (3) No reliable change was defined as a BDI-II score neither increased above, nor reduced by more than 8 points. (4) Deteriorated was defined as a BDI-II increase above 8 and a total score above 14. Data analyses were performed with SPSS version 23 (IBM SPSS Statistics 23 [44]).

#### Power Analysis

As described in the study protocol [30], the study had a power of 0.97 to detect an effect size of *d*=0.50 or higher with a sample size of N=230.

#### Randomization

As delineated in the study by Zwerenz et al [28], randomization (intervention group and control group) was performed using the software Research Randomizer [45] by the Study Center of Mental Disorders of the University Medical Center Mainz (block randomization at a ratio of 1:1) faxing the assignment to the study assistant at the clinic, who performed the assignment, accordingly.

## Results

### Primary and Secondary Outcomes

Table 1 reports means and SDs of the observed scores at baseline and the estimated scores at follow-up (T3) for the primary and secondary outcomes.

**Figure 1:**
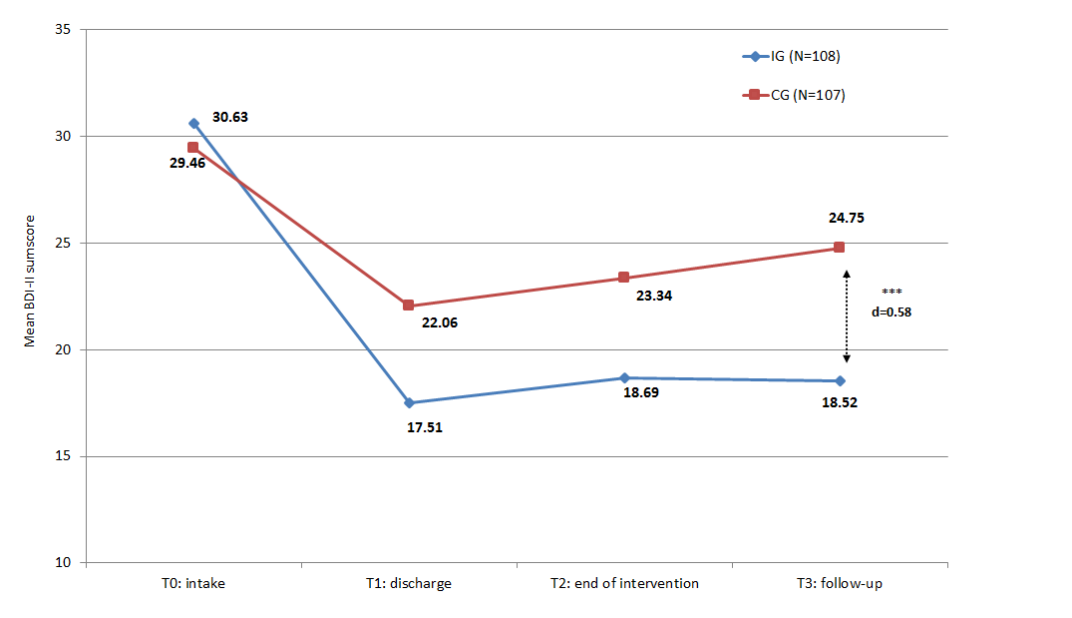
Primary outcome (BDI-II) in the course of time. BDI-II: Beck Depression Inventory II; CG: control group; IG: intervention group; T0: allocation to intervention (baseline); T3: follow-up 6 months after baseline.

At follow-up (T3), intervention group and control group significantly improved regarding depressive symptoms assessed with the BDI-II (Table 1). Within-group effect size was high in the intervention group and moderate in the control group. The between-group comparison showed a medium effect size (*P*<.001; *d*=0.58). As can also be seen in Figure 1, the intervention group shows at any point in time after T0 a stronger reduction in the BDI-II than the control group.

Table 2 reports test statistics as well as effect sizes for the primary and secondary outcomes at follow-up (T3) after 6 months compared with study intake (T0).

**Table 1:** Descriptive statistics of primary and secondary outcomes at 6-month follow-up (T3) of intervention (N=108) and control group (N=107), compared with study admission (T0).

Outcome criteria		Baseline (T0^a^), mean (SD)	Follow-Up (T3^b^), mean^c^ (SD)
**BDI-II^**d**^**			
	IG^e^	30.63 (9.39)	18.52 (10.78)
	CG^f^	29.46 (9.50)	24.75 (10.74)
**PHQ-9^g^**			
	IG	14.86 (4.88)	10.37 (5.54)
	CG	14.30 (5.23)	13.13 (5.70)
**GAD-7^h^**			
	IG	11.59 (4.28)	8.24 (4.68)
	CG	11.57 (5.08)	10.38 (4.68)
**EUROHIS^i^**			
	IG	1.62 (0.57)	2.13 (0.68)
	CG	1.68 (0.59)	1.84 (0.66)
**RSE^j^**			
	IG	15.11 (6.68)	17.78 (6.60)
	CG	15.40 (6.80)	15.75 (6.60)
**DAS^k^**			
	IG	149.96 (37.05)	139.78 (34.26)
	CG	158.42 (38.26)	145.82 (34.78)
**WAI^l^**			
	IG	25.41 (2.87)	26.47 (3.73)
	CG	25.28 (2.60)	26.30 (4.55)

^a^T0: allocation to intervention (baseline).

^b^T3: follow-up 6 months after baseline.

^c^Estimated means.

^d^BDI-II: Beck Depression Inventory.

^e^IG: intervention group.

^f^CG: control group.

^g^PHQ-9: Patient Health Questionnaire-9.

^h^GAD-7: Generalized Anxiety Disorder-7.

^i^EUROHIS: European Health Interview Survey.

^j^RSE: Rosenberg Self-Esteem Scale.

^k^DAS: Dysfunctional Attitude Scale.

^l^WAI: Work Ability Index.

There were no significant differences at baseline (T0) between intervention group and control group for any of the secondary outcome measures. A lower symptom load at follow-up (T3) in the intervention group in comparison with the control group could also be observed for the secondary outcomes. Analyses revealed statistically significant between group differences and low-to-moderate effect sizes for depressive symptoms assessed with the PHQ-9 (*P*<.001; *d*=0.49), anxiety (GAD-7; *P*<.001; *d*=0.46); quality of life (EUROHIS-QOL 8; *P*=.002; *d*=0.43), and self-esteem measured by the RSE scale (*P*=.02; *d*=0.31). There were no significant differences comparing both groups concerning dysfunctional attitudes assessed with the DAS (*P*=.34; *d*=0.18) and work ability (WAI; *P*=.45; *d*=0.04). Furthermore, within-group comparisons showed that both groups benefitted not only regarding depressive symptoms measured by the PHQ-9 with an effect size of *d*=0.80 in the intervention group and *d*=0.22 in the control group but also for anxiety (intervention group: *d*=0.69, control group: *d*=0.24) and quality of life (intervention group: *d*=0.71, control group: *d*=0.25).

**Table 2:** Between-group and within-group comparisons for primary and secondary outcomes.

Outcome criteria		T3^a^			T0^b^-T3		
		Between-group comparisons^c^			Within-group comparisons^d^		
		*F* (*df*)	*P* value^e^	*d*	*t* (*df*)*; r*	*P* value	*d*
**BDI-II^**f**^**		18.87 (1,212)	<.001	0.58	—^g^	—	—
	IG^h^	—	—	—	10.21 (1665); 0.42	<.001	1.06
	CG^i^	—	—	—	4.94 (1671); 0.57	<.001	0.44
**PHQ-9^**j**^**		13.733 (1,212)	<.001	0.49	—	—	—
	IG	—	—	—	7.488 (13863); 0.38	<.001	0.80
	CG	—	—	—	2.136 (1088); 0.42	<.001	0.23
**GAD-7^**k**^**		12.802 (1,212)	<.001	0.46	—	—	—
	IG	—	—	—	6.497 (6851); 0.39	<.001	0.69
	CG	—	—	—	2.252 (19067); 0.38	<.001	0.24
**EUROHIS^**l**^**		9.557 (1,212)	<.001	0.43	—	—	—
	IG	—	—	—	6.895 (1095); 0.43	<.001	0.71
	CG	—	—	—	2.488 (866); 0.48	<.001	0.25
**RSE^**m**^**		5.427 (1,212)	.02	0.31	—	—	—
	IG	—	—	—	3.585 (7165); 0.44	<.001	0.37
	CG	—	—	—	0.589 (4437), 0.45	.55	0.06
**DAS^**n**^**		0.955 (1,212)	.34	0.18	—	—	—
	IG	—	—	—	3.917 (34211); 0.62	<.001	0.33
	CG	—	—	—	2.558 (7731); 0.52	.01	0.24
**WAI^**o**^**		0.676 (1,212)	.45	0.04	—	—	—
	IG	—	—	—	2.558 (99); 0.22	.01	0.31
	CG	—	—	—	1.948 (31); 0.04	.06	0.26

^a^T3: Follow-up 6 months after baseline.

^b^T0: Allocation to intervention (baseline).

^c^Analyses of covariance with baseline as covariate.

^d^Paired samples *t* tests.

^e^Level of significance .05.

^f^BDI-II: Beck Depression Inventory II.

^g^Not applicable.

^h^IG: intervention group.

^i^CG: control group.

^j^PHQ-9: Patient Health Questionnaire-9.

^k^GAD-7: Generalized Anxiety Disorde-7.

^l^EUROHIS: European Health Interview Survey.

^m^RSE: Rosenberg Self-Esteem Scale.

^n^DAS: Dysfunctional Attitude Scale.

^o^WAI: Work Ability Index.

### Remission, Improvement, and Deterioration

**Figure 2:**
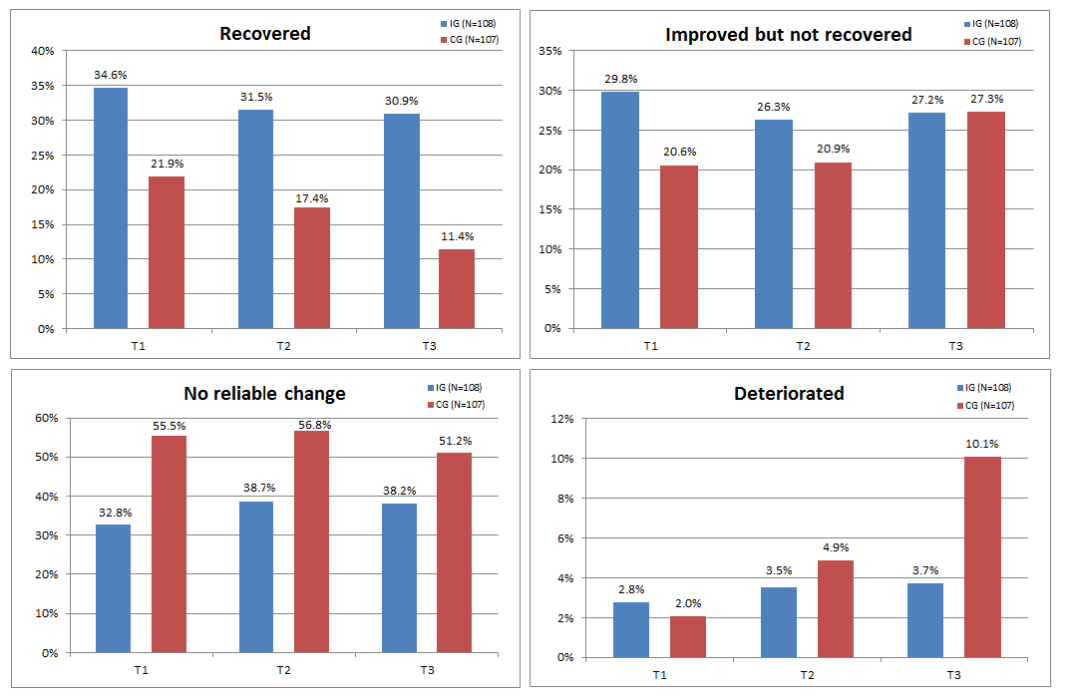
Recovery, improvement, and deterioration in the course of time. BDI-II: Beck Depression Inventory II; CG: control group; IG: intervention group; T1: discharge; T2: end of intervention; T3: follow-up 6 months after baseline.

Comparisons between intervention group and control group are shown in Figure 2. As can be seen in Figure 2, the rate of participants experiencing a remission or an improvement was significantly higher in the intervention group than in the control group at each point of measurement (T1: χ^2^_3_=11.5; *P*=.01; *d*=0.36; T2: χ^2^_3_=9.3; *P*=.03; *d*=0.31; T3: χ^2^_3_=15.2; *P*=.002; *d*=0.55). The gap even widened over time with an NNT of 7.84 at discharge (T1), 7.09 at the end of intervention (T2), and 5.12 at follow-up (T3).

### Predictors of Depressive Symptoms at Follow-Up 6 Months Later

Following the study protocol, multivariate analyses were performed to test the effects of postulated predictors on depressive symptoms at follow-up 6 months later (T3). These were depressive symptoms at study intake and at discharge, utilization of other treatments at follow-up, childhood trauma, and structural characteristics. GLM (R²=0.688; *F*_118,200_=4.813; *P*<.04, *η*_*p*_^*2*^=0.040) based on imputed data revealed that depressive symptoms at discharge from hospital treatment was the strongest predictor (Table 3). Interestingly, baseline depressive symptoms played no additional role. Group membership (Deprexis vs control group) was an additional predictor of depressive symptoms at follow-up, and there was a trend to an interaction between depressive symptoms at discharge and group. Other variables which had been posited in our study protocol as potential predictors (psychotherapeutic treatment at follow-up, childhood trauma, structural characteristics) as well as sex and age played no role as predictors.

**Table 3:** Predictors of depressive symptoms at 6-month follow-up (T3) based on generalized linear model (N=200).

Predictors	*F* (*df*)	*P* value^a^	*η_p_^2^* ^b^
Age	0.460 (1)	.25	0.004
Sex	0.316 (1)	.28	0.003
CTQ^c^ at T0^d^	0.991 (1)	.16	0.008
OPD-SFK^e^ at T0	0.454 (1)	.25	0.004
Utilization of other treatments at follow-up	0.294 (1)	.30	0.002
BDI-II^f^ at T0	1.285 (1)	.26	0.011
BDI-II at T1^g^	2.630 (46)	<.001	0.506
Group	3.243 (1)	.04	0.027
BDI-II at T1 X Group	1.267 (28)	.10	0.231

^a^Level of significance .05, 1-tailed test.

^b^Partial eta square.

^c^CTQ: Childhood Trauma Questionnaire.

^d^T0: Study admission (baseline).

^e^OPD-SFK: Operationalized Psychodynamic Diagnosis.

^f^BDI-II: Beck Depression Inventory.

^g^T1: Discharge of inpatient treatment.

## Discussion

### Principal Findings

Following our first publication to the efficacy of Deprexis offered as an add-on to inpatient psychotherapy [28], this study investigated effects on the course of depressive symptoms of the Web-based self-management program 6 months after study inclusion. Participation in the adjunct Web-based self-management program improved the course of depressive symptoms during the follow-up period. The gap of effectiveness between the intervention group and the control group, which received access to Web-based information about depression and depressive symptoms in addition to inpatient psychotherapy, even widened over the course of the study. This was reflected in an increase of the between group effect size from *d*=0.44 to *d*=0.58. When we differentiated between different categories of outcome, the proportion of remission of 35% in the Deprexis group at discharge was almost maintained at follow-up (31%), whereas remission in the control group declined from 22% to 11% in the same period. Comparable proportions of patients achieved substantial improvements but did not fulfill criteria of remission (about 27%) at follow-up. More than twice as many participants (10% vs 4%) deteriorated in the control group compared with the intervention group 6 months after study inclusion. Correspondingly, the NNT improved in favor of the intervention group (from 7.84 at discharge from inpatient treatment to 5.12 at follow-up). This result, that 1 in every 5 to 8 patients benefitted from the program, is not only comparable with other studies on self-guided interventions [46] but also with results from face-to-face therapies [47].

Additional and moderate improvements were found in Deprexis compared with the control group regarding the PHQ-9 measure of depressive symptoms, generalized anxiety (GAD-7), and quality of life (EUROHIS-QOL), all of which also improved in the control group. Self-esteem (RSE) only improved moderately in the intervention group but not the control group. Dysfunctional attitudes (DAS) improved in both groups, whereas WAI was significant in the intervention (trend in the control group), and there were no differential benefits.

Consistent with previous studies [26], residual depressive symptoms at termination of inpatient treatment were the strongest predictor of depressive symptoms at follow-up, in addition to a small group effect in favor of the intervention. Baseline scores of depressive symptoms, childhood trauma, psychic structure, and demographic characteristics played no additional role. Thus, superiority of the intervention versus the control condition rests mainly on the fact that the intervention reduces residual depressive symptoms compared with controls. As indicated by the trend to an interaction, continued use of the program may further reduce depressive symptoms during follow-up. However, this is a very small effect at best.

Inpatient treatment usually applies to complex cases of depression with different mental and somatic comorbidities [48]. Resonating with other findings [26], inpatient psychotherapy should strive not only to improve depressive symptoms scores (among other goals) but also to reduce residual depressive symptoms further. The relationship of symptom reduction and duration, respectively, dose of treatment is considered complex because of different patient trajectories of change and therapist influences [49]. Compared with 61.8 days in a multicenter study conducted by Franz et al [23] from 2007 to 2011 and 10 weeks in the previous trial of Zeek et al [25], the total duration of 40 days of inpatient treatment was relatively short. Cutting down treatment duration in the medical system to save cost may lead to higher rates of relapse or recurrence.

### Strengths and Limitations

The high effects of our adjunct to inpatient multimodal psychotherapy are consistent with a recent review, in which evidence was shown that Web-based interventions are efficacious for maintaining treatment gains and prevent relapse [50]. This leads to the question, what barriers have to be overcome and what are the challenges for integrating new technologies and interventions into an existing treatment setting, such as, for example, low acceptance of Web-based interventions in the treatment team [51,52], technological barriers, or low acceptance and compliance within patients [53].

In view of chronic trajectories of depressive symptoms, the short follow-up interval of about 3 months (mean 96.59 days, SD 20.21 days) following termination of the program must be considered a drawback. However, our data underline that even the brief time period of the first months after discharge sets the stage for deterioration for many patients—notwithstanding continued outpatient care. Although the study raises important economic issues, health economic data were not collected. Another limitation is that we mainly relied on self-reports and have no clinical diagnostics after discharge from inpatient treatment. Furthermore, we do not know exactly what kind of treatment and what treatment intensity patients received after discharge from the hospital and of course this could have an influence on the course of depressive symptoms. But after all, we could control for the 1 item that asked if any kind of treatment followed inpatient treatment (yes/no), which was not a significant predictor of depressive symptoms at follow-up.

Although the Web-based self-help program Deprexis has been demonstrated to be efficacious [11], the challenge is how to integrate it into regular mental health care. To our knowledge, we were the first to compare Deprexis as an add-on to inpatient psychotherapy to an active control group receiving Web-based psychoeducational information in an RCT. Compliance and retention were very good with dropout rates between 3% and 4% between the different timepoints of the study and no differences in dropout rates between intervention group and control group [28]. We, therefore, presume that a structured inpatient treatment format is suitable for adding adjunct Web-based self-help. However, as blended therapy is currently regarded as promising to improve psychotherapeutic treatment, the question arises how implementation could take place and what challenges have to be overcome. In a qualitative study of therapist’s perspective on blended psychotherapy for depression [54], the most frequent barriers reported were limited customizability and autonomy of decisions concerning blended treatment, disease-related contraindications, negative affect because of technical problems, the limitation of face-to-face sessions as a consequence of blending the therapy, or the impairment of therapeutic alliance because of technical problems. Facilitators were also mentioned but less frequently, such as, motivation and willingness of patients for innovative interventions, the possibility for patients to use Web-based contents between face-to-face sessions, or the contemporary treatment possibility which could also close the treatment gap [54].

### Conclusions

Tapping into self-help resources of patients by adding Web-based self-help is a promising way to improve long-term outcomes. There is a need for more studies, but if other studies also conclude that Web-based interventions as an add-on or blended treatment are effective, the challenge will be to implement them in the health care system so that reimbursement is possible. An ongoing development, regular updating, and securing of the latest technical standards are associated with corresponding costs. Therefore, it must be investigated who can bear these costs and how much they are. However, if that succeeds, efficacious Web-based interventions could be implemented on a broader basis to improve the benefits and sustainability of face-to-face treatments.

## Abbreviations

BDI-II: Beck Depression Inventory-II
DAS: Dysfunctional Attitude Scale
EUROHIS-QOL 8: European Health Interview Survey Quality of Life 8-Item Index
GAD-7: generalized anxiety disorder-7
GLM: generalized linear model
NNT: number needed to treat
PHQ-9: Patient Health Questionnaire-9
RCI: Reliable Change Index
RCT: randomized controlled trial
RSE: Rosenberg Self-Esteem Scale
TAU: Treatment as Usual
WAI: Work Ability Index
